# Helpful and challenging aspects of breastfeeding in public for women living in Australia, Ireland and Sweden: a cross-sectional study

**DOI:** 10.1186/s13006-020-00281-0

**Published:** 2020-05-12

**Authors:** Yvonne L. Hauck, Lesley Kuliukas, Louise Gallagher, Vivienne Brady, Charlotta Dykes, Christine Rubertsson

**Affiliations:** 1 Department of Nursing and Midwifery Education and Research, King Edward Memorial Hospital, Perth, Western Australia Australia; 2 School of Nursing, Midwifery and Paramedicine, Curtin University, Perth, Western Australia Australia; 3 School of Nursing and Midwifery, Trinity College Dublin, Dublin, D02 T283 Ireland; 4 Skåne University Hospital, Lund, Sweden; 5 Institute of Health Sciences, Medical Faculty, Lund University, Lund, Sweden

**Keywords:** Breastfeeding in public, Decision-making, Mothers, Experiences

## Abstract

**Background:**

Breastfeeding in public continues to be contentious with qualitative evidence confirming that women face many challenges. It is therefore important to gain understanding of not only the challenges but also what women perceive is helpful to breastfeed in public.

**Methods:**

A cross-sectional study was conducted with women living in Australia, Ireland or Sweden currently breastfeeding or having breastfed within the previous 2 years. Our objective was to explore and compare what women do when faced with having to breastfeed in the presence of someone they are uncomfortable with and what women think is helpful and challenging when considering whether to breastfeed in public. Data were collected in 2018 from an online survey over a 4 week period in each country. Content analysis revealed data similarity and theme names and definitions were negotiated until consensus was reached. How often each theme was cited was counted to report frequencies. Helpful and challenging aspects were also ranked by women to allow international comparison.

**Results:**

Ten themes emerged around women facing someone they were uncomfortable to breastfeed in the presence of with the most frequently cited being: ‘made the effort to be discreet’; ‘moved to a private location’; ‘turned away’ and ‘just got on with breastfeeding’. Nine themes captured challenges to breastfeed in public with the following ranked in the top five across countries: ‘unwanted attention’; ‘no comfortable place to sit’; ‘environment not suitable’; ‘awkward audience’ and ‘not wearing appropriate clothing’. Nine themes revealed what was helpful to breastfeed in public with the top five: ‘supportive network’; ‘quiet private suitable environment’; ‘comfortable seating’; ‘understanding and acceptance of others’ and ‘seeing other mothers’ breastfeed’.

**Conclusions:**

When breastfeeding in public women are challenged by shared concerns around unwanted attention, coping with an awkward audience and unsuitable environments. Women want to feel comfortable when breastfeeding in a public space. How women respond to situations where they are uncomfortable is counterproductive to what they share would be helpful, namely seeing other mothers breastfeed. Themes reveal issues beyond the control of the individual and highlight how the support required by breastfeeding women is a public health responsibility.

## Background

The importance of breastfeeding is indisputable and comprehensively supported through recommendations from the World Health Organization (WHO) and United Nations Children’s Fund [1, 2]. A recent UNICEF analysis from 123 countries highlighted that 95% of all babies received some breastmilk. However, rates vary between countries with 4% of babies from low and middle income countries never receiving breastmilk increasing to 21% for babies from high income countries [1]. Variations exist within high income countries with almost 98% of Swedish babies and 92% of Australian babies receiving breastmilk whereas 55% of Irish babies are breastfed [1].

Research has revealed challenges around women’s experience of breastfeeding, with one challenge being the management of breastfeeding in public. High income countries such as Sweden, Australia, New Zealand, England, and the United States have provided qualitative evidence on women’s experiences of breastfeeding in public. Although most research explored women’s experiences with breastfeeding in general, a recurring theme around the challenges of breastfeeding in public has consistently emerged.

An interpretative phenomenological study with five Australian mothers’ journey of exclusive breastfeeding to 6 months, revealed shared commonalities related to the difficulties with public breastfeeding with an emphasis on the sexuality of breasts [3]. To achieve exclusive breastfeeding to 6 months, those who could not overcome their awkwardness with the practice offered expressed breastmilk in a bottle to reach their goal [4]. Another recent Australian study explored views and beliefs of first time breastfeeding mothers and members of their social network and analysed nine family conversations involving 50 participants [5]. Participant views were that women were required to be “discreet and covered to not expose the breast, select an appropriate place to avoid discomforting others, guard against judgement and protect herself from unwanted male gaze” [5]. These common challenges experienced when breastfeeding in public were present and heightened for overweight and obese Swedish [6], American [7] and New Zealand women [8] due to larger breasts and difficulties with latching their babies, which made being discreet problematic and feeling ashamed and self-conscious due to exposure.

Research has identified other groups of breastfeeding women with heightened vulnerability around public breastfeeding which includes African immigrants and refugees. The breastfeeding experiences of 31 African refugee women living in Australia revealed how women perceived the lack of visibility of public breastfeeding contributed to stigma and shame [9]. Difficulties and being discouraged to breastfeed in public were also noted by 15 African American women who shared issues around sustaining their breastfeeding [10] and 22 African American mothers who felt stigmatised based upon ‘spectators’ actions’ [11].

Additional vulnerable groups included young mothers and those living in a country where legal protection to breastfeed in public was limited. An Australian study of 24 young (17 to 25 years of age) mothers focused upon their need for information and support, and found that when breastfeeding in public they felt more exposed as a ‘young mum’ which brought more attention to them: any attention was noted as predominantly negative [12]. Even British women who are legally protected to breastfeed in public for up to 6 months, but chose to breastfeed longer than 6 months, experienced suspicion and disapproval [13]. Eight women in this qualitative study shared how they experienced ‘really horrible looks’, ‘stigma from families and community’ and ‘feeling quite exposed’ (p.231).

The influence of perceived cultural norms around breastfeeding in public was also highlighted during interviews with 27 Chinese mothers, who expressed their struggle and embarrassment, given that exposing breasts in their culture was considered unacceptable and ‘uncivilized’ [14]. Women in Ireland (*n* = 7) who shared experiences of being in a public health nurse led support group, valued being in a breastfeeding group that felt like a ‘cocoon of normality’ in a infant formula feeding culture, where breastfeeding was something to be ashamed of [15].

Research highlighting the experiences of women breastfeeding in public provides insight into this phenomenon whilst exploring breastfeeding experiences in general. Findings have been generated from qualitative research capturing the stories of breastfeeding women across international contexts and have predominately emphasised the negative or challenging aspects of the experience. Although breastfeeding rates to 6 months differ between Sweden (72%), Australia (60%) [16] and Ireland (26 to 29%) [17, 18], similar categories were revealed when women from these countries shared what assisted them to breastfeed [19]. Informal face to face support and maternal determination ranked within the top five categories across the three countries [19]. Although regarding breastfeeding as a cultural norm was cited by women living in Australia, Ireland and Sweden, this category was ranked eight out of ten and focused upon the importance of having a strong family history of breastfeeding and having this reinforced with key role models. The issue of managing breastfeeding in public was not acknowledged.

The challenges around breastfeeding in public have been recognised in qualitative studies with small numbers of participants from high income countries. Women’s perceptions of what is helpful when considering whether to breastfeed in public have not been researched and presents a gap in knowledge. Greater insight into what breastfeeding women perceive can help and challenge them can inform initiatives and schemes to counter the challenges and enhance factors to better support their efforts to breastfeed in public. Due to the variation in breastfeeding across high income countries we chose two countries with high initiation rates (Australia and Sweden) and a third with lower rates (Ireland) [1]. Our international study focused on exploring and comparing what women from three high income countries perceive as helpful or challenging when breastfeeding in public, in addition to revealing what they do when faced with having to breastfeed in the presence of someone they are uncomfortable with.

## Methods

A cross-sectional study was undertaken using an online survey with women living in Australia, Ireland and Sweden. Ethical approval to conduct the study was granted by Curtin University Human Research Ethics Committee in Australia (HRE2018–0037), Research and Ethics Committee, School of Nursing and Midwifery, Trinity College in Ireland (COM_35_17/18) and the Advisory Committee for Research Ethics in Health Education Lund University in Sweden (Reference Number 50–18). Women who were living in Australia, Ireland or Sweden and were currently breastfeeding or had breastfed within the previous 2 years were invited to participate in the online survey through social media.

Three specific research questions were posed: 1) What do women do when they are faced with having to breastfeed in front of someone they are uncomfortable with, 2) What do women think was most helpful when considering whether to breastfeed in public and 3) What do women think was most challenging when considering whether to breastfeed in public? Women were then asked to rank their responses to questions 2) and 3) as first, second or third in importance based upon what they felt were the most helpful and challenging aspects. The online survey was presented on a user-friendly platform suitable for completion on a mobile phone (Qualtrix in Australia, SurveyMonkey in Ireland and Sweden). In addition to the three open ended questions noted above, demographic data on maternal age, education level, number of children / number of children ever breastfed, and whether they were still breastfeeding their youngest child at the time of survey completion was collected.

A poster with a link to the online survey was developed for each country and with university approval circulated through social media. The initial screen was an information letter which included the inclusion criteria. Women had to confirm that they were living in the specified country, met the inclusion criteria and consented to participate prior to accessing the online survey. The original poster was included in a maternity consumer Facebook page in Australia that encouraged women to share with other mothers and within 24 h, over 2600 women had completed the online survey. In Sweden the translated poster was included in several Facebook pages mainly with parental interest such as Home parents network, Public breastfeeding, The breastfeeding help group, Baby slings and Close parenting. In Ireland a link to an online survey was posted on popular Breastfeeding support pages on Facebook. The post provided study details and clicking the link brought women to an information page where they consented to participate before commencing the survey.

Data were collected over a four-week period in 2018 in each country (March in Australia, April in Ireland and December in Sweden). Originally, we anticipated leaving the online survey open for 3 months but due to the overwhelming response from women in all three countries, data collection was ceased after a four-week period.

Responses to the three open ended questions provided rich data that was analysed using content analysis, which is often used with textual data from open-ended survey questions [20]. Responses to each question were exported from the online platforms into separate Word documents for analysis by investigators in each country. Content analysis involves description at a surface level around individual shared experiences presented in participants’ own words [21]. A systematic coding and categorising approach was conducted with this textual information to determine common themes [22]. Content analysis was undertaken by reviewing all responses from women who responded and continued until data saturation became apparent. Initial thematic analysis was conducted separately by two investigators in each country. Tentative themes from data in each country were then shared amongst the international team and it became apparent that themes were more similar than different. Therefore, to facilitate comparison between breastfeeding women living in the three countries, we negotiated final theme names and definitions until a consensus agreement was reached.

Once the final themes were confirmed between countries, we were then able to consider the possibility of counting how often each theme was cited and reported frequencies using descriptive statistics. Due to the numbers of women responding and after themes were confirmed separate SPSS databases were developed and data were then manually entered under each theme. This process facilitated counting how often each theme was cited and ranked as first, second or third in relation to being helpful or challenging when considering breastfeeding in public. At this stage all responses from women in Ireland and Sweden were entered to determine frequencies. As the Australian sample was up to four times larger, a systematic process to collect a representative sample (every second or third entry) was adopted. Statistics were only chosen to describe frequencies of the themes as our intention was not to make inferences or generalizations between women in each country.

## Results

Survey respondents included 10,910 women living in Australia, 1835 living in Ireland and 1520 living in Sweden. A summary of characteristics such as age, education level, parity, number of children ever breastfed and whether they were still breastfeeding when completing the survey are presented in Table 1. The mean age of women living in Ireland was older (34.9 years) compared to women in Australia (32.3 years) or Sweden (32.8 years). The sample reflects women with a high level of education as 89.2% of Irish women had a university or college degree compared to 82.4% from Sweden and 70.6% from Australia. The majority (83.0%) of women living in Australia were born in Australia with 8.4% born in Europe. The majority (81.7%) of women living in Ireland were born in Ireland with 13.7% born in Europe. The majority (93%) of women living in Sweden were born in Sweden with 6% born in Europe.

**Table 1:** Demographic characteristics of women living in Australia, Ireland and Sweden

**Demographic Characteristics**	**Women living in Australia**	**Women living in Ireland**	**Women living in Sweden**
Maternal age (*N *= 10,885 Australia, *N *= 1831 Ireland, *N *= 1520 Sweden)	32.3 mean	34.9 mean	32.8 mean
(*N* = 10,885 Australia,	32 median	35 median	32 median
*N* = 1831 Ireland,	33 mode	35 mode	32 mode
*N* = 1520 Sweden)	Range 17 to 53 (SD 4.79)	Range 18 to 49 (SD 4.109)	Range 18 to 57 (SD 4.92)
Highest education level (*N* = 10,910 Australia, *N* = 1820 Ireland, *N* = 1520 Sweden)			
University / college postgraduate degree	2997 (27.5%)	943 (51.8%)	835 (55.0%)
University / college undergraduate degree	4707 (43.1%)	681 (37.4%)	417 (27.4%)
High / secondary school completed	2623 (24.0%)	183 (10.1%)	252 (16.6%)
High / secondary school not completed	583 (5.3%)	13 (0.7%)	16 (1%)
Number of children (*N* = 10,910 Australia, *N* = 1800 Ireland, *N* = 1409 Sweden)			
1 child	4990 (45.7%)	761 (41.9%)	677 (48.0%)
2 children	4097 (37.6%)	664 (36.6%)	537 (38.0%)
3 children	1341 (12.3%)	226 (15.0%)	150 (11%)
4 or more children	482 (4.4%)	109 (6.4%)	45 (3%)
Number children ever breastfed (*N* = 10,910 Australia, *N* = 1835 Ireland, *N* = 1410 Sweden)			
1 child	5225 (47.9%)	828 (45.1%)	707 (50.2%)
2 children	4024 (36.9%)	671 (36.6%)	523 (37.1%)
3 children	1245 (11.4%)	249 (13.6%)	138 (9.8)
4 or more children	416 (3.8%)	86 (4.7%)	41 (2.9%)
Still breastfeeding youngest child (*N* = 10,910 Australia, *N* = 1837 Ireland, *N* = 1443 Sweden)	7607 (69.7%)	1288 (70.1%)	950 (62.5%)

### Faced with having to breastfeed in front of someone they are uncomfortable with

A total of 4742 women living in Australia, 1139 living in Ireland and 1348 living in Sweden responded to this question. Content analysis revealed ten themes commonly cited by women from all three countries. The themes in no particular order were: Made the effort to be discreet; Moved to a private location; Turned away; Just got on with breastfeeding; Never felt uncomfortable; Not my problem; Flagged their intention to breastfeed; Tried to avoid the situation; Used expressed breastmilk (EBM) or infant formula and Had someone supportive with them. A definition of each theme with supporting quotes from women in Australia, Ireland and Sweden is provided in Table 2.

**Table 2:** Themes capturing what women did when faced with having to breastfeed in front of someone they are uncomfortable with

**Theme & Definition**	**Supporting quotes from women**
**Made the effort to be discreet** Used an array of devices to cover the breast and minimise exposure such as a muslin wrap, shawls, feeding apron or particular clothing	• *Used a muslin cloth to cover up [Aus]* • *Just try and be as discreet and covered up as possible [Aus]* • *I put a muslin cloth over me to hide anything that might become visible when my baby stopped [Irish]* • *Tried to be discreet,* i.e. *used a scarf or jacket to cover baby’s head. Asked if it they minded if I breastfed my baby [Irish]* • *Tried to not show my nipple by turning away or cower myself with clothes until the baby was latched on and started suckling. Then adjust my clothes so as little as possible of my nipple was showing [Swe]* • *Tried to hurry up my baby to latch on quickly because I did want to show my nipple. Then I covered up both of us immediately [Swe]*
**Moved to a private location** Physically moved to private location such as another room, a car or a public toilet/bathroom	• *Went to another room or the car [Aus]* • *Breastfed in car instead of public. Moved to somewhere more private [Aus]* • *I would have left the room if possible to breastfeed or went to a toilet or the car [Irish]* • *Leave the room [Irish]* • *Moved to another room if we were in other’s home. Tried to find a private place if we were outdoors [Swe]* • *Placed myself and baby further away from people staring [Swe]*
**Turned away** Turned or positioned body to not be facing the other person particularly during vulnerable period of exposure when attaching and detaching baby	• *I usually just turn away from the person while I get the baby attached and then turn back [Aus]* • *I would either turn so my side/back was facing them [Aus]* • *I tried to sit sideways so that if my baby unlatched I could cover myself quickly [Irish]* • *I would turn my back until baby latched on then made sure my breast was fully covered [Irish]* • *Never been so uncomfortable that I did not breastfed, but turned away so my back was towards the uncomfortable people [Swe]* • *Turned away to be more in privacy, at least when he latched on [Swe]*
**Just got on with breastfeeding** Baby was the focus and their needs were paramount no matter and took no notice of others	• *Concentrated on my baby [Aus]* • *I just looked at my beautiful baby and reminded myself it was all about him, not the other person [Aus]* • *I fed anyway. Baby’s needs take priority [Irish]* • *I would think of my baby’s need to feed and stop other thoughts and get on with it [Irish]* • *Kept focusing on my child, told myself that it is us (baby and me) who counts, others can go away. Felt defiant, protecting my baby, and also safe in my relation to my child. Told myself that if I stand up for myself and my baby, in the long run my baby will stand up for herself (building up her self-confidence) [Swe]* • *Tried to get over that bad feeling and breastfeed anyway, my baby’s needs comes first. But I also worked out strategies and readymade answers to be able to defend us [Swe]*
**Never felt uncomfortable breastfeeding in front of other people** Did not experience a situation where they felt uncomfortable	• *Have never felt that uncomfortable [Aus]* • *I have never felt so uncomfortable that it has been a problem [Aus]* • *I have never felt uncomfortable breastfeeding in front of anyone [Irish]* • *Never occurred - no reason to be uncomfortable [Irish]* • *I am not uncomfortable in front of anyone, when my baby needs I am breastfeeding [Swe]* • *Never felt uncomfortable, carried on, prioritized the need of my child first, realized that I am breaking norms [Swe]*
**Not my problem** Some also felt defiance in the stance they took and either asked the other person to leave, or if they were uncomfortable suggested they can decide to leave	• *I just feed. Always. It was not my child’s problem or mine if anyone else was uncomfortable [Aus]* • *It’s my child’s right to feed when they want to. It’s natural. If other people feel uncomfortable, that’s not my problem [Aus]* • *Got on with it telling myself that their discomfort is theirs...not mine to take on [Irish]* • *I breastfed. My baby, my rules. If someone has a problem, then that’s their issue. Not my concern [Irish]* • *Tried to think that breastfeeding is super natural and if the person has problems with me breastfeeding then that’s their problem and I must do the best for my child [Swe]* • *I feel confident breastfeeding, something quite natural, whenever my baby needs it. If anyone else has a problem with breastfeeding it is their problem and nothing that I will have an impact on me [Swe]*
**Flagged their intention to breastfeed** Some women either apologised, asked if they minded or warned others of their intention to breastfeed	• *I warned them I was going to breastfeed and then proceeded to do so [Aus]* • *Warned them I was going to breastfeed now. If they felt uncomfortable they could come back when I was done [Aus]* • *I just said, “hope you don’t mind, I’ve to feed baby” [Irish]* • *Said what I was about to do and smiled [Irish]* • *Asked and convinced myself that the person was okay with me breastfeeding [Swe]* • *I asked about permission to breastfed my baby in front of them [Swe]*
**Tried to avoid the situation** Used strategies to avoid being put in a situation by monitoring the timing of feeds or delaying a feed until they were in a comfortable environment	• *Try to console baby without feeding until I was not around a person who made me uncomfortable [Aus]* • *Tried to delay the feed and wait until was somewhere I felt comfortable [Aus]* • *Held off for as long as possible,* i.e. *distracted/ dummy, if had then fed very discreetly with use of blanket [Irish]* • *I delayed feeding due to comfort level [Irish]* • *Tried to delay the feeding to later, if possible [Swe]* • *Distracted my baby to delay the feeding [Swe]*
**Expressed breastmilk or infant formula as backup** Had a bottle of expressed breast milk or infant formula as a backup should a situation arise where they were uncomfortable to feed	• *I would pump so I could use bottles in such situations [Aus]* • *I always had bottles of formula with me in case these moments came [Aus]* • *Sometimes brought a bottle of expressed milk [Irish]* • *If I knew situation would arise that a feed was due when in company I had a bottle with me [Irish]* • *In certain situations I chose the bottle instead of breastfeeding – it was mostly for practical reasons due to that I wanted nice clothes (up dressed) [Swe]* • *Expressed milk in advance and gave it from a bottle in such special situations [Swe]*
**Had someone supportive with them** Women tried to have someone with them who was supportive and comfortable to advocate for their need to breastfeed	• *I’d do it if I had to, I’d likely get my husband or a female friend to sit between us [Aus]* • *I have been lucky that when people, usually strangers, have an issue that I have been with others that support my feeding and make me comfortable [Aus]* • *Ideally have my partner for moral support and to deal in case anything was said, nothing ever was but I sometimes got hurtful looks [Irish]* • *It really helped to be with supportive people [Irish]* • *I was hiding behind my partner [Swe]* • *My partner supported me and told them that our baby needs breastfeeding [Swe]*

All responses from women in Ireland (*N* = 1139) and Sweden (*N* = 1348) were entered into a statistical package to determine frequencies for each response being cited by women from each country. To achieve a number for comparison with Irish and Swedish data, every third response was entered from Australia data (*N* = 1600). ‘Made the effort to be discreet’ was cited most frequently across all countries (62.1% in Australia; 44.9% in Ireland; 38.1% in Sweden). The frequencies for each theme are presented in an approximate descending order in Table 3 and were comparable across each country. However, one theme ‘Just got on with breastfeeding’ ranked third for women in Australia (22.8%) and Sweden (20.0%) and fourth at 9.0% for Irish women. Use of expressed breast milk (EBM) or infant formula was ninth for Australian women (1.4%) and Irish women (1.1%) but equal tenth for Swedish women (0.04%).

**Table 3:** What women do when faced with having to breastfeed in front of someone they are uncomfortable with

**Themes**	**Women living in Australia** **(*N*** **= 1600)** ***n* (%)**	**Women living in Ireland** **(*N*** **= 1139)** ***n* (%)**	**Women living in Sweden** **(*N*** **= 1348)** ***n* (%)**
Made the effort to be discreet	994 (62.1%)	512 (44.9%)	513 (38.1%)
Moved to a private location	454 (28.4%)	291 (25.5%)	272 (20.2%)
Turned away	217 (19.0%)	334 (20.9%)	154 (11.4%)
Just got on with breastfeeding	364 (22.8%)	103 (9.0%)	270 (20.0%)
Never felt uncomfortable breastfeeding in front of others	108 (6.8%)	82 (7.2%)	62 (4.6%)
Not my problem	77 (4.8%)	18 (1.6%)	14 (1.0%)
Flagged their intention to breastfeed	62 (3.9%)	41 (3.5%)	8 (0.6%)
Tried to avoid the situation	57 (3.6%)	34 (2.9%)	44 (3.3%)
Used EBM or infant formula	22 (1.4%)	13 (1.1%)	6 (0.4%)
Had someone supportive with them	13 (0.8%)	7 (0.6%)	5 (0.4%)

Women offered multiple responses so frequencies do not add up to 100%

### Challenges to breastfeeding in public

A total of 4596 women living in Australia, 1648 living in Ireland and 1220 living in Sweden provided a response to what was challenging about breastfeeding in public. Content analysis revealed nine themes commonly cited by women across the three countries. The themes were: Unwanted attention; No comfortable place to sit; Environment not suitable; Awkward audience, Not wearing appropriate clothing; Potential for baby to be distracted; Struggling with feeding issues; Managing a distressed baby and Having to manage other children. A definition of each theme with supporting quotes from women in Australia, Ireland and Sweden is provided in Table 4.

**Table 4:** What was challenging and helpful around breastfeeding in public

**Challenging**		**Helpful**	
**Theme & Definition**	**Supporting quotes from women**	**Theme & Definition**	
**Unwanted attention** Includes ‘fear of’ unwanted attention as well as ‘actual’ unwanted attention, blatant and overt staring, being judged, disapproving comments especially when breastfeeding an older infant / toddler	• *Worrying about judgemental looks from others and that someone will say something [Aus]* • *Fear of someone commenting and then getting upset [Aus]* • *Fear of someone saying something to you - never happened to me in over 5 years total feeding but we’ve all heard stories or seen inflammatory stories in magazines, online etc about mothers being verbally abused for feeding [Irish]* • *Fear of disapproval and being confronted - although I didn’t encounter it [Irish]* • *The risk of being confronted while having to show my breasts [Swe]* • *People do have a lot to say about breastfeeding and they do speak out giving comments. I don’t want that attention [Swe]*	**Supportive network** Having a supportive person with you such as a partner, family member or friend AND having access to online peer support around breastfeeding in public	• *My partner’s support and whoever else I may be with [Aus]* • *Reassurance – having a support person the first few times, if someone said something negative they could respond and I could focus on feeding and they can remind me it’s okay to be feeding [Aus]* • *Support from partner, friends, family, public health nurse [Irish]* • *Being part of an online community of breastfeeding mothers [Irish]* • *Company that supports you and reassures you [Swe]* • *Understanding people around you [Swe]*
**No comfortable place to sit** A comfortable chair is not available	• *Somewhere to sit [Aus]* • *Lack of appropriate seating [Aus]* • *A physically comfortable place to nurse [Irish]* • *Finding suitable seating especially when I don’t necessarily want to pay for the privilege, go to a café [Irish]* • *I needed an arm chair and support for my back [Swe]* • *None or very uncomfortable seating [Swe]*	**Quiet private suitable environment** Designated parents room that is clean, has water available, able to contain older children, can accommodate a pram / stroller, changing facilities, not smelly, suitable temperature, shaded if outside, noise level [quiet & non-distracting]	• *Having a quiet / private place to go [Aus]* • *More parks with fences for toddlers so you can breastfeed [Aus]* • *I look for a breastfeeding friendly sign on door [Irish]* • *Somewhere private or enclosed [Irish]* • *To be able to find a nice place not in other people’s sight [Swe]* • *A special public place set aside [Swe]*
**Environment not suitable** The environment could be perceived to be noisy, unclean, smelly, crowded, people were smoking, there was no privacy, the temperature inside or outside being too hot or cold and there was no room for a stroller / pram	• *Being outside in undesirable weather [Aus]* • *Noisy busy environment [Aus]* • *There were hardly any feeding rooms anywhere [Irish]* • *Facilities, some places have awful feeding rooms, they stink, overflowing nappy bins in them, or no rooms or chairs or anywhere where you can sit quickly and feed [Irish]* • *The weather, too cold [Swe]* • *Crowded, noisy and stressful environment [Swe]*	**Comfortable seating** Chair with a supportive back & armrests	• *Comfy chairs! [Aus]* • *Somewhere comfortable to sit [Aus]* • *Comfortable seating area [Irish]* • *Somewhere to sit down [Irish]* • *Comfortable chair [Swe]* • *A nice bench to be able to sit outside [Swe]*
**Awkward audience** This audience included men, teenagers, older generation who are perceived as disapproving, other mothers infant formula feeding in parents room, or religious setting such as a place of worship	• *Older generation looking uncomfortably at me [Aus]* • *Lots of men / strangers around [Aus}* • *Attitude of Irish public to breastfeeding (has improved but still some backward views) [Irish]* • *If there are male teenagers about, I would find it slightly more uncomfortable. Age demographic in general- would also be a little bit more wary in front of older people [Irish]* • *Older generation with a different opinion about breastfeeding [Swe]* • *Young men or teenagers [Swe]*	**Understanding and acceptance of others** Receiving smiles, encouragement & reassurance from general public; offers to help with pram, toddler or getting refreshments such as water	• *General acceptance within the community [Aus]* • *Strangers who would say ‘good on you’ when feeding in public [Aus]* • *Waiting staff being helpful* e.g. *topping up my tea, getting water etc [Irish]* • *Pleasant non-intrusive encouragement [Irish]* • *It would be helpful if others showed a more positive attitude to breastfeeding [Swe]* • *A warm accepting smile from others and NO comments [Swe]*
**Not wearing appropriate clothing** If the woman was not in appropriate clothing to allow breastfeeding discreetly or did not bring a suitable cover	• *Making sure I dress in a way that is easy to breastfeeding without exposing too much [Aus]* • *How accessible my breast is; what clothing I have worn [Aus]* • *Clothing and self-image. I wouldn’t care about BF would it would bother me if I didn’t have my stomach covered [Irish]* • *Exposing my tummy is a worry for me if I have the wrong clothes on [Irish]* • *To always have to think about appropriate clothing in advance to be able to breastfeed [Swe]* • *Wearing “the right” clothes without exposing too much [Swe]*	**Seeing other mothers breastfeed** Seeing other mothers breastfeed is supportive & helps to normalise breastfeeding	• *Seeing other mums breastfeeding with confidence [Aus]* • *Being surrounded by other mothers who also feed wherever they need to [Aus]* • *Normalisation of feeding - seeing others feeding [Irish]* • *Sisters and cousins normalised it for me, once I saw them I was more confident to go ahead in public too [Irish]* • *A mother friendly accepting culture, positive response and lots of mothers breastfeeding in public spaces [Swe]* • *Seeing other mothers breastfeeding gives confidence [Swe]*
**Potential for baby to be distracted** This applied particularly to older infant who more aware of surroundings and could come off and on the breast to look at the distraction	• *When baby started becoming more alert to surrounds and was more easily distracted in public [Aus]* • *Having an older child that is easily distracted and comes on and off frequently [Aus]* • *As baby gets older and they become nosey I’d want the place not to be too busy so as to limit [Irish]* • *My toddler being free to roam as I had the baby tethered to me, that was the only issue I ever had, chasing the toddler and then expose myself [Irish]* • *My baby is too curious, comes on and off too often [Swe]* • *My baby gets distracted and had difficulties attaching [Swe]*	**Wearing suitable clothing or having a cover** Having suitable clothing and/or a cover/wrap to facilitate being able to breastfeed discreetly if that is desired	• *Feeding clothes designed for feeding are essential for me [Aus]* • *Breastfeeding tops without clips make it easier to feed in public and to get my breast out [Aus]* • *Wearing two tops. So a vest could cover my belly and the other top could be pulled up so minimum skin is shown [Irish]* • *Breast feeding apron [Irish]* • *Comfy clothes to not show the nipples [Swe]* • *Nice clothes designed for breastfeeding. You want to be able to dress nicely as a mother! [Swe]*
**Struggling with feeding issues** Struggling with feeding issues such as using a nipple shield, positioning, attachment issues, or reflux	• *Putting nipple shield on, then latching baby [Aus]* • *I have a strong let down and it can go everywhere or I leak from the opposite side [Aus]* • *When baby is young and we are both getting to grips with BF-ing it can be difficult with latching baby on, leakage, sore nipples etc while trying not to expose myself [Irish]* • *I had fast let down so it would mean the baby would cough and spitter, so I would have to take him off the breast and milk would spray out! It put me off feeding in public for a little while until I was able to manage it better [Irish]* • *From the beginning when the milk was all over, very embarrassing [Swe]* • *Struggling with the nipple shield [Swe]*	**Preparation and confidence** Preparation and confidence referred to situation where the mother breastfed previously, or received timely support to gain skills and build confidence	• *Support from a lactation consultant to feed properly which helped with my overall confidence in feeding [Aus]* • *Midwife to suggest nipple shields to be able to breastfeed in the first place [Aus]* • *Having bit of practice at home in the first few days/weeks so baby latches easily enough and more confidence [Irish]* • *Booklet from maternity hospital or public health nurse on how to adapt your clothes for feeding what works and hacks no need for nursing cloths all of the time as regular cloths can be adapted and can be a lot more discreet at times too [Irish]* • *Myself being confident breastfeeding [Swe]* • *To do it with self-confidence even if it was a little complicated (outside my comfort zone)[Swe]*
**Managing a distressed baby** The mother has to manage a baby who is distressed due to hunger or impatience	• *How hungry my son is [Aus]* • *How fussy my baby is being [Aus]* • *If baby is crying [Irish]* • *Having an over hungry/crying baby drawing attention to it [Irish}* • *A fussy baby and all the mess [Swe]* • *Shame if the baby is crying and fussy [Swe]*	**Breastfeeding is overtly welcomed** Signage; welcome stickers displayed or breastfeeding / family friendly venues	• *Having a sign saying breastfeeding accepted [Aus]* • *Sign saying breastfeeding welcome [Aus]* • *Parent baby movie screenings, a great way to get out and about with a new baby and bf in public for first time without feeling “watched”. [Irish]* • *Sign saying breastfeeding welcome here, such a small thing but makes a big difference [Irish]* • *A sticker “You are welcome to breastfeed here”[Swe]* • *A sign saying****Breastfeeding welcome here****that you can point out if someone gives you negative comments [Swe]*
**Having to manage other children** Not having a safe environment to contain other children within the mother’s sight	• *Having something to contain and entertain my 2 year old while I’m tied down [Aus]* • *Making sure my 3 year old is safe and entertained while feeding his brother [Aus]* • *Managing a toddler at the same time [Irish]* • *Toddler running wild when outside the home and it was hard to manage if feeding [Irish]* • *To be able to handle siblings simultaneously [Swe]* • *Difficult when alone, taking care of more than one child [Swe]*	**Knowledge of mother’s and children’s rights** Knowing you have rights as a breastfeeding mother and are protected by law	• *Knowledge of my rights to breastfeed whenever, wherever my baby want to [Aus]* • *Knowledge of the law and breastfeeding! New mothers need to know their breastfeeding rights [Aus]* • *The law [Irish]* • *Educate staff that breastfeeding is completely legal anywhere [Irish]* • *Children’s rights to get food when they need [Swe]* • *Legal rights to breastfeed in public [Swe]*

Women were asked to rank their responses to challenges as first, second or third to reflect their importance (Table 5). All responses from women in Ireland (*n* = 1648) and Sweden (*n* = 1220) were entered into a statistical package to determine frequencies and to achieve a number for comparison, every second response was entered from Australia data (*n* = 2332). ‘Unwanted attention’ was ranked as first by women in Australia (27.0%) and Sweden (38.2%) with the ‘environment not suitable’ ranked highest by women in Ireland (29.0%) with ‘unwanted attention’ marginally lower at 28.3%. ‘No comfortable place to sit’ was ranked second most frequently (20.1%) by Australian women, third (17.0%) by Irish women and fourth (6.6%) by Swedish women. “Having to manage other children’ was ranked lowest across the countries (1.9, 1.0, 0.2%)”.

**Table 5:** Most challenging and helpful themes ranked as first

**Challenging themes**	**Women living in Australia** **(*N*** **= 2332)** ***n*(%)**	**Women living in Ireland** **(*N*** **= 1648)** ***n*(%)**	**Women living in Sweden** **(*N =*** **1220)** ***n*(%)**
Unwanted attention	629 (27.0%)	467 (28.3%)	466 (38.2%)
No comfortable place to sit	468 (20.1%)	281 (17.0%)	80 (6.6%)
Environment not suitable	439 (18.8%)	479 (29.0%)	213 (17.5%)
Awkward audience	281 (12.0%)	318 (19.2%)	220 (18.0%)
Not wearing appropriate clothing	174 (7.5%)	71 (4.3%)	16 (1.3%)
Potential for baby to be distracted	122 (5.2%)	97 (5.8%)	9 (0.7%)
Struggling with feeding issues	111 (4.8%)	48 (2.9%)	37 (3.0%)
Managing a distressed baby	63 (2.7%)	21 (1.2%)	9 (0.7%)
Having to manage other children	45 (1.9%)	17 (1.0%)	2 (0.2%)
**Helpful themes**	**Women living in Australia** **(*N*** **= 2466)** ***n*(%)**	**Women living in Ireland** **(*N*** **= 1744)** ***n*(%)**	**Women living in Sweden** **(*N*** **= 1250)** ***n*(%)**
Supportive network	459 (18.6%)	500 (28.7%)	114 (9.1%)
Quiet private suitable environment	450 (18.2%)	166 (9.5%)	231 (18.5%)
Comfortable seating	387 (15.7%)	169 (9.7%)	167 (13.4%)
Understanding and acceptance of others	377 (15.3%)	286 (16.4%)	321 (25.7%)
Seeing other mothers breastfeed	203 (8.2%)	206 (11.8%)	126 (10.1%)
Wearing suitable clothing or having a cover	200 (8.1%)	72 (4.1%)	56 (4.5%)
Preparation and confidence	170 (6.9%)	176 (10.1%)	84 (6.7%)
Breastfeeding is overtly welcomed	154 (6.2%)	145 (8.3%)	71 (5.7%)
Knowledge of mother’s and children’s rights	66 (2.7%)	24 (1.4%)	85 (6.8%)

Women offered multiple responses so frequencies do not add up to 100%

One point of difference around perceived challenges noted in the data involved comments around sexism and was expressed by 90 women in Sweden. Examples of comments included: *Peoples view on breasts, the sexism of breasts, how men look at you while breastfeeding, to get “undressed” in public and the feeling of being “naked” while breastfeeding.* This terminology was not found in comments from women in Ireland or Australia and therefore was not considered a common theme.

### Helpful to breastfeeding in public

A total of 4924 women living in Australia, 1744 living in Ireland and 1250 living in Sweden provided a response to what was helpful when considering whether to breastfeed in public. Content analysis revealed nine themes commonly cited by women across the three countries. The themes were: Supportive network; Quiet private suitable environment; Comfortable seating; Understanding and acceptance of others; Seeing other mothers breastfeed; Wearing suitable clothing or having a cover; Preparation and confidence; Breastfeeding is overtly welcomed; and Knowledge of mother’s and children’s rights. A definition of each theme with supporting quotes from women in all three countries is provided in Table 4.

In a similar process to the challenges, women were asked to rank their responses to what was helpful as first, second or third (Table 5). All responses from women in Ireland (*n* = 1744) and Sweden (*n* = 1250) and every second response from Australia data (*n* = 2466) were entered into a statistical package to determine frequencies for each response from women from each country. ‘Supportive network’ was ranked highest by women in Australia (18.6%) and Ireland (28.7%). ‘Understanding and acceptance of others’ ranked highest in Sweden (25.7%), and was second in Ireland (16.4%) and fourth in Australia (15.3%). ‘Seeing other mothers breastfeed’ was comparable across countries (8.2, 11.8, 10.1%) and ‘Knowledge of rights’ ranked lowest in Australia and Ireland (2.7 and 1.4%) whereas ‘Wearing suitable clothing or having a cover’ ranked lowest in Sweden (4.5%).

## Discussion

In summary, although ten themes emerged around strategies women used when facing someone they were uncomfortable to breastfeed in the presence of, the four most frequently cited were: ‘made the effort to be discreet’; ‘moved to a private location’; ‘turned away’ and ‘just got on with breastfeeding’. Nine themes captured challenges to breastfeed in public with ‘unwanted attention’ ranked highest for women in Australia and Sweden whereas ‘environment not suitable’ ranked highest for women in Ireland. Nine themes addressed what was helpful to breastfeed in public and ‘supportive network’ ranked highest for women in Australia and Ireland compared to ‘quiet private suitable environment’ for women in Sweden. ‘Seeing other mothers breastfeed’ ranked in the top five across all three countries.

Challenges to breastfeeding in public identified in this study are supported by previous research. Concepts noted in qualitative studies from seven countries focused on exposure of the body, feeling uncomfortable and vulnerable, embarrassment, perceived lack of acceptability, fear of confrontation or actual negative reactions, positioning challenges, being discreet, and minimising feeding around a particular audience. This synopsis of concepts reflects study findings from Australia [23], Ireland [15], Sweden [6], the United States [7], China [14], Canada [24] and the United Kingdom [25, 26]. However, our findings offer further information around additional challenges mothers considered when deciding to breastfeed in public such as the ‘suitability of the environment’, ‘having a comfortable place to sit’, the ‘potential for the baby to be distracted’, ‘managing a distressed baby’, and ‘having to manage other children’. Innovative strategies to address these challenges warrant attention. One example that could be shared and replicated to other local contexts includes the use of technology. A recent study analysed reviews by British mothers on a ‘FeedFinder App’ which involved women sharing information on facilities, service, level of privacy and venue quality to assist decisions around where to breastfeed in public [27]. This innovation where women assist other women offers an empowering strategy which has implications for business owners as they would be motivated to ensure their facility was positively rated to boost rather than negatively impact their customer base.

Themes that were captured as enablers represent a novel approach with potential modifiable findings to enhance women’s experience of breastfeeding in public. Findings provide insight into areas to target changes and improvements to better support breastfeeding mothers which aligns with the salutogenic model and what contributes to the promotion and maintenance of health. Women’s sense of coherence delineated in the salutogenic model has been applied to infant feeding experiences using constructs such as comprehensibility (belief in understanding challenges), manageability (having sufficient resources) and meaningfulness (desire to respond to challenge) [28].

Some themes were the reverse of the challenges such as having a ‘supportive network’, the ‘understanding and acceptance of others’, a ‘quiet private suitable environment’, ‘comfortable seating’ and ‘having clothing or a cover’ if the mother chooses to be discreet. Having a supportive network extends beyond face to face contact to locating support through social media. A qualitative study of exclusive breastfeeding with 30 New Zealand mothers found that smartphone apps are a genuine option for promoting breastfeeding. Information is accessed and shared on Facebook amongst breastfeeding mothers who may not have a close relationship but are encouraged by a sense of community [29]. Even closed Facebook groups such as those hosted by the Australian Breastfeeding Association have been found to provide not only informational but emotional support to breastfeeding women [30]. Other themes identified in this international study highlight strategies that could be further promoted, such as signage to ‘overtly welcome breastfeeding mothers’, improving opportunities for mothers to ‘see other mothers breastfeeding’, and ensuring women and the general community are aware of ‘mothers’ and ‘children’s’ rights.

Specific themes in our findings address issues that are beyond the control of the individual and require addressing public attitudes to breastfeeding, so women encounter supportive networks that demonstrate understanding and acceptance of breastfeeding in public. Recognition that breastfeeding is a public health responsibility has been supported by evidence suggesting the barriers to breastfeeding extend beyond the individual mother to societal influences [31]. Increasing awareness of mothers’ and children’s rights around breastfeeding may present a necessary tactic to inform and educate the public of the importance of breastfeeding to our society and our future generations. Signage that overtly welcomes breastfeeding, that provides an environment with comfortable seating, gives a message that endorses a culture where breastfeeding is seen as ‘normal’. Community involvement is essential for any change process as illustrated in a recent inquiry approach to discover how communities can better support breastfeeding. Community conversation workshops were recently undertaken with Australian parents, grandparents, children’s services, local government, health services and retail owners [32]. Two related themes were revealed acknowledging the importance of PLACE: ‘sometimes a PLACE to breastfeed’ and ‘the parent room: a hidden and unsafe PLACE to breastfeed’. Although characteristics of communities that provided a ‘sanctuary’ were noted, the participants indicated that breastfeeding in public was rarely observed which supports the theme ‘seeing other mothers breastfeed’ as being an important enabling theme.

‘Made the effort to be discreet’ was the most frequently cited strategy that women across Australia, Ireland and Sweden noted when faced with having to breastfeed in front of someone they are uncomfortable with. Other themes included ‘moving to a private location’, ‘turning away’, and ‘tried to avoid the situation’ which are all activities counterproductive to the helpful theme of wanting to ‘see other mothers breastfeed’. A recent discussion paper on the requirement to justify breastfeeding in public suggests that if breastfeeding could be recognised as a “family way of life” and positive interaction this stance would support a “more right to breastfeeding in public without social sanction, whether one is able to breastfeed discreetly or not” [33].

Facilitating a culture where breastfeeding in public is acceptable and women feel comfortable require initiatives to address societal attitudes given that ‘unwanted attention’ and ‘awkward audience’ also ranked within the top four themes negatively influencing a decision to breastfeed in public. Interventions to change societal attitudes have met some success and highlight strategies that can be shared internationally. For example, a prime-time television clip depicting public breastfeeding decreased the extent to which American students believed breastfeeding should be private while improving attitudes and support for breastfeeding in public [34]. Use of a music video parody with young Canadian adults found that shorter intervals between seeing the video and shorter intervals between follow-up ratings measured increasing comfort levels thereby reinforcing the importance of being exposed to breastfeeding [35]. Finally, another study included use of posters to enhance comfort levels with members of the Canadian public surveyed at shopping centers and found that only 51.9% were comfortable with a woman breastfeeding anywhere in public but this increased to 84.5% in a doctor’s office or 81.4% in a park [36]. There were significant improvements in comfort levels following viewing of promotional posters depicting breastfeeding in a doctor’s office and restaurant.

The influence of seeing a woman breastfeed extends beyond being an enabler to existing breastfeeding women and can influence the decision whether to breastfeed at all. A systematic review focused on factors influencing expectant parents infant feeding choice revealed a bias towards negative factors relating to the breastfeeding decision as parents went through a process of weighing reasons for and against breastfeeding [37]. Factors influencing choice of infant feeding method included female role models, family and support network and feeding in front of others/public. Expectant parents are already aware and thinking about how they could manage breastfeeding in public [37]. A longitudinal study with 259 Scottish first time mothers who had never breastfed and agreed with a statement that ‘it was lovely to see her breastfeed’ were six times more likely to intend to breastfeed [38].

Coordinated action and targeted strategies must consider all factors to address the challenges women experience when breastfeeding in public. To facilitate the promotion and support of breastfeeding, all influencing factors must be acknowledged within an ecological model, such as the mother and infant dyad, the family, the health care system, the community and societal and culture factors [39]. Breastfeeding represents a complex experience. Results from a systematic review and meta-analysis recommended that multicomponent interventions be implemented across the childbirth continuum to include antenatal and postnatal periods, hospital and community contexts and the involvement of health professionals [40]. A multifaceted approach where support needs to be layered was recommended in a Health Promotion Strategic Framework in Australia to include mothers, family and community [41]. Awareness of this approach is apparent in the Australian National Breastfeeding Strategy that endorses the need for a social marketing campaign targeting specific audiences to change attitudes and behaviours regarded as unsupportive of breastfeeding [42].

More women from Sweden than Australia or Ireland shared comments about sexualization of the female body, the ‘male gaze’, and a feeling of being attributed to a sex object when breastfeeding. Without existing evidence, we cannot speculate the reason for the differences between countries and why Swedish women were more aware and outspoken about sexism. Therefore additional international research is warranted around this issue to further explore how awareness and expression of this concern may differ depending upon a particular context or culture.

Limitations to our international findings must acknowledge the demographic characteristics of the international samples. The women who responded were recruited through social media and therefore are connected to social networks; whether this connection is supportive or not cannot be determined. The sample strongly represents highly educated women, who are in their thirties with one child living in high income countries with varying breastfeeding initiation and prevalence rates. Participant characteristics were not unexpected as populations recognised as being more at risk of not breastfeeding include women under 25 years of age, with low socio-economic status, and low education levels [42]. Strategies to target hard to reach participants for research also acknowledge younger women and reasons cited for not completing assessments which include lack of time and loss of interest [43]. However, the response rate, achieved within 4 weeks of releasing the survey, does highlight the importance and passion that women feel toward the topic of breastfeeding in public, particularly in the Australian context.

## Conclusions

The most challenging theme for mothers in Australia and in Sweden were ‘unwanted attention’ and for mothers in Ireland it was ‘environment not suitable. Women have indicated that a feeling of awkwardness and discomfort discourages breastfeeding in public. A ‘supportive network’ was cited as most helpful for women in Australia and Ireland with ‘understanding and acceptance of others’ being more helpful for women in Sweden. Breastfeeding women want to feel comfortable, accepted, supported and welcomed. Understanding the issues that help and challenge breastfeeding in public and addressing the concerns women have raised in this international study is one step forward. Encouraging and welcoming women to breastfeed in public may be one strategy that is helpful to women and promotes societal change to norms where it is hidden and an uncommon sight.

## Data Availability

The datasets generated and analysed during the current study are not publicly available as they are not all in English and were not combined but are available from the corresponding author on reasonable request.
